# *trans*-*rac*-[1-Oxo-2-phenethyl-3-(2-thien­yl)-1,2,3,4-tetra­hydro­isoquinolin-4-yl]methyl 4-methyl­benzene­sulfonate

**DOI:** 10.1107/S1600536808029309

**Published:** 2008-09-17

**Authors:** Mehmet Akkurt, Sema Öztürk Yıldırım, Milen G. Bogdanov, Meglena I. Kandinska, Orhan Büyükgüngör

**Affiliations:** a Department of Physics, Faculty of Arts and Sciences, Erciyes University, 38039 Kayseri, Turkey; b Faculty of Chemistry, University of Sofia, 1, James Bourchier blvd., 1164 Sofia, Bulgaria; c Department of Physics, Faculty of Arts and Sciences, Ondokuz Mayıs University, 55139 Samsun, Turkey

## Abstract

The title compound, C_29_H_27_NO_4_S_2_, was synthesized by reaction of *trans*-*rac*-4-(hydroxy­meth­yl)-2-phenethyl-3-(thio­phen-2-yl)-3,4-dihydro­isoquinolin-1(2*H*)-one and 4-methyl­benzene-1-sulfonyl chloride in the presence of Et_3_N in CH_2_Cl_2_. The relative orientations of the benzene ring (*A*) of the 3,4-dihydro­isoquinolinone ring system, the thio­phene ring (*B*), the benzene ring (*C*) of the methyl­benzene group and the phenyl ring (*D*) result in the following dihedral angles: *A*/*B* = 80.91 (16), *A*/*C* = 22.79 (18), *A*/*D* = 9.9 (2), *B*/*C* = 80.73 (19), *B*/*D* = 88.9 (2) and *C*/*D* = 29.9 (2)°. The crystal structure is stabilized by weak inter­molecular C—H⋯O hydrogen bonds and C—H⋯π inter­actions.

## Related literature

For chemical background, see: Kandinska *et al.* (2006 ▶); Rothweiler *et al.* (2008 ▶). For ring puckering parameters, see: Cremer & Pople (1975 ▶).
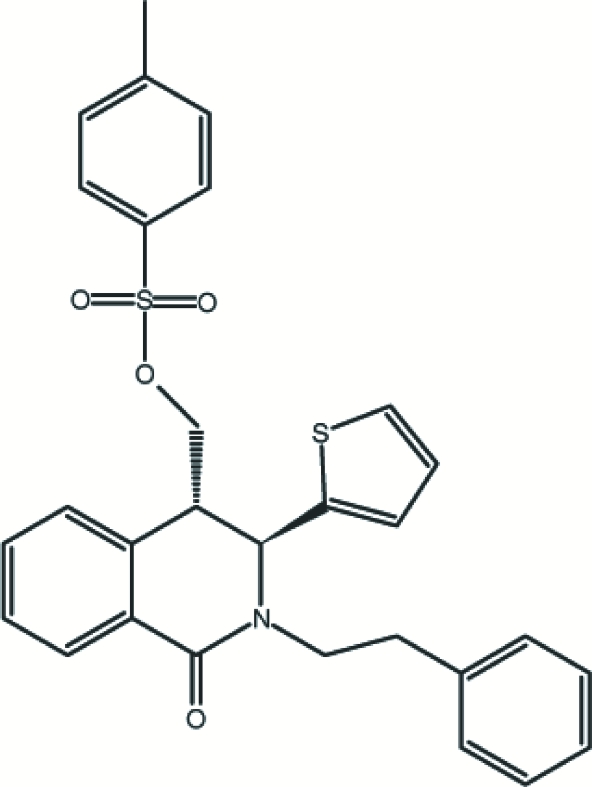


## Experimental

### 

#### Crystal data

C_29_H_27_NO_4_S_2_
*M* *_r_* = 517.66
Triclinic, 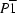

*a* = 7.2529 (3) Å
*b* = 8.6727 (4) Å
*c* = 20.9899 (10) Å
α = 86.021 (4)°
β = 87.396 (4)°
γ = 78.330 (4)°
*V* = 1289.24 (10) Å^3^
*Z* = 2
Mo *K*α radiation
μ = 0.24 mm^−1^
*T* = 293 K
0.58 × 0.52 × 0.48 mm

#### Data collection

STOE IPDS 2 diffractometer
Absorption correction: integration (*X-RED32*; Stoe & Cie, 2002 ▶) *T*_min_ = 0.872, *T*_max_ = 0.892
24485 measured reflections
5417 independent reflections
4537 reflections with *I* > 2σ(*I*)
*R*_int_ = 0.026

#### Refinement

*R*[*F* ^2^ > 2σ(*F* ^2^)] = 0.090
*wR*(*F* ^2^) = 0.306
*S* = 1.36
5417 reflections
328 parameters
H-atom parameters constrained
Δρ_max_ = 2.04 e Å^−3^
Δρ_min_ = −1.13 e Å^−3^

### 

Data collection: *X-AREA* (Stoe & Cie, 2002 ▶); cell refinement: *X-AREA*; data reduction: *X-RED32* (Stoe & Cie, 2002 ▶); program(s) used to solve structure: *SIR97* (Altomare *et al.*, 1999 ▶); program(s) used to refine structure: *SHELXL97* (Sheldrick, 2008 ▶); molecular graphics: *ORTEP-3 for Windows* (Farrugia, 1997 ▶); software used to prepare material for publication: *WinGX* (Farrugia, 1999 ▶).

## Supplementary Material

Crystal structure: contains datablocks global, I. DOI: 10.1107/S1600536808029309/hb2793sup1.cif

Structure factors: contains datablocks I. DOI: 10.1107/S1600536808029309/hb2793Isup2.hkl

Additional supplementary materials:  crystallographic information; 3D view; checkCIF report

## Figures and Tables

**Table 1:** Hydrogen-bond geometry (Å, °)

*D*—H⋯*A*	*D*—H	H⋯*A*	*D*⋯*A*	*D*—H⋯*A*
C9—H9⋯O4^i^	0.98	2.34	3.285 (3)	161
C12—H12⋯*Cg*1^ii^	0.93	2.65	3.567 (4)	168

Symmetry codes: (i) 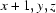; (ii) 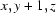. *Cg*1 is the centroid of the thiophene ring (S2/C18–C21).

**Fig. 1.**
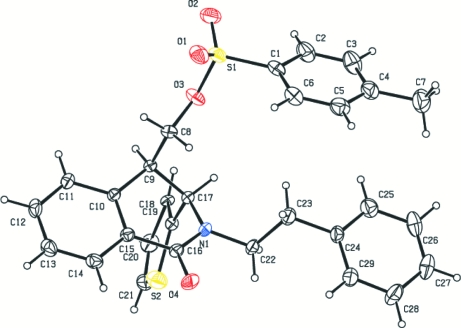
The molecular structure of (I), with 20% probability displacement ellipsoids for the non-hydrogen atoms.

**Fig. 2.**
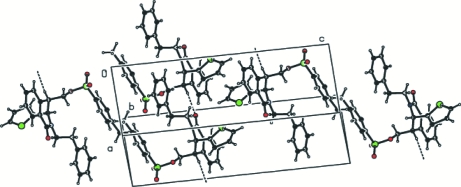
View of the packing and C—H···O4(x + 1, y, z) hydrogen bonding interactions of (I).

## supplementary crystallographic information

### Comment

The title compound, (I), was synthesized as part of a research project (Kandinska *et al.*,  2006) seeking precursors for the production of new tetrahydroquinolone derivatives with biological activity (Rothweiler *et al.*,  2008).

In the molecule of (I) (Fig.1), the benzene ring A (C10—C15) of 3,4-dihydroisoquinolinone ring system is essentially planar, with an r.m.s. deviation of 0.005 (3) Å for C11 and its other six-membered part is not planar [its Puckering parameters (Cremer & Pople, 1975) are Q_T_ = 0.444 (3) Å, θ = 118.4 (4) ° and φ = 93.4 (4) °]. The thiophene ring B (S2—C18—C21) is almost planar, with an r.m.s. deviation for fitted atoms of 0.004 Å. The rings C (C1—C6) and D (C24—C29) are almost planar, with an r.m.s. deviation for fitted atoms of 0.012 Å and 0.021 Å, respectively. The dihedral angles between the planes of these rings are A/B = 80.91 (16), A/C = 22.79 (18), A/D = 9.9 (2), B/C = 80.73 (19), B/D = 88.9 (2) and C/D = 29.9 (2)°. An interesting feature of the crystal structure is the long C18—C19 bond of 1.594 (3) Å.

The crystal structure of (I) is stabilized by weak intra- and intermolecular C—H···O hydrogen bonds and C—H···π interactions (Table 1 and Fig. 2).

### Experimental

Compound(I) was synthesized by reaction between *trans*-*rac*-4-(hydroxymethyl)-2-phenethyl-3-(thiophen-2-yl)-3,4-\ dihydroisoquinolin-1(2*H*)-one (5.91 g, 0.0163 mol) and 4-methylbenzene-1-sulfonyl chloride (6.22 g, 0.033 mol) in the presence of Et_3_N (6.8 ml, 0.049 mol) in CH_2_Cl_2_. After working up the reaction mixture, compound (I) crystallized as colourless prisms from hexane–ethyl acetate (9:1 v/v) (yield 7.5 g, 89%; m.p. 385–386 K). Elemental analysis, calculated for C_29_H_27_NO_4_S_2_: C 67.29, H 5.26°; found: C  66.90, H 5.45°. IR (KBr) 3000 cm^-1^ (C—H), 1647 cm-1 (C=O), 1603 cm-1 (ArH), 1467 cm^-1^ (ArH), 1358 cm^-1^ (S=O), 1172 cm^-1^ (S=O). ^1^H NMR (250 MHz, CDCl_3_) δ (p.p.m.) = 2.32 (s, 3H, *Ph—C*H*_3_*), 2.69 (t, 2H, J = 8.3 Hz, *Ph—C*H*_2_*), 3.05–3.17 (m, 1H, *N—CH_2_^a^*), 3.45–3.54 (ddd, 1H, J = 1.3, 5.1 and 10 Hz, *-OCH_2_—C*H), 3.69 (t, 1H, J = 10.3 Hz, *-SO_3_—CH_2_^a^*), 4.04–4.23 (m, 2H, *-SO_3_—C*H*_2_^b ^*& *N—C*H*_2_^b^*), 5.10 (d, 1H, J = 0.8 Hz, *Th—CH*), 6.81 (dd, 1H, J = 3.5 and 4.9 Hz, *Th-*H), 6.76–6.81 (m, 1H, *Ph-*H), 7.06 (dd, 1H, J = 1.4 and 4.9 Hz, *Th-*H), 7.10–7.16 (m, 1H, *Ph-*H), 7.17–7.23 (m, 3H, *H*-Ph), 7.24–7.28 (m, 2H, *Th-*H, *Ph-*H), 7.28–7.33 (m, 2H, *Ph-*H), 7.40–7.48 (m, 2H, *Ph-*H), 7.78 (d, 2H, J = 8.3, *Ph-*H), 8.08–8.15 (m, 1H, *Ph-*H). ^13^C NMR (63 MHz, CDCl_3_) δ = 162.8, 145.4, 142.7, 138.4,133.4, 132.6, 132.4, 130.1, 129.0, 128.8, 128.7, 128.5, 128.4, 127.8, 126.5,126.4, 125.6, 125.1, 70.0, 56.8, 48.5, 45.8, 34.1, 21.5.

### Refinement

The H atoms were positioned geometrically, with C—H = 0.93-0.97 Å, and refined using a riding model, with *U*_iso_(H) = 1.2 or 1.5*U*_eq_(C). The maximum diference peak and deepest difference hole are situated 0.13 Å from C19 and 0.36 Å from S2, respectively.

### Crystal data

**Table:**

C_29_H_27_NO_4_S_2_	*Z* = 2
*M**_r_* = 517.66	*F*(000) = 544
Triclinic, *P*1	*D*_x_ = 1.334 Mg m^−^^3^
Hall symbol: -P 1	Mo *K*α radiation, λ = 0.71073 Å
*a* = 7.2529 (3) Å	Cell parameters from 39940 reflections
*b* = 8.6727 (4) Å	θ = 2.0–27.2°
*c* = 20.9899 (10) Å	µ = 0.24 mm^−^^1^
α = 86.021 (4)°	*T* = 293 K
β = 87.396 (4)°	Prism, colourless
γ = 78.330 (4)°	0.58 × 0.52 × 0.48 mm
*V* = 1289.24 (10) Å^3^	

### Data collection

**Table:**

STOE IPDS 2 diffractometer	5417 independent reflections
Radiation source: sealed X-ray tube, 12 x 0.4 mm long-fine focus	4537 reflections with *I* > 2σ(*I*)
plane graphite	*R*_int_ = 0.026
Detector resolution: 6.67 pixels mm^-1^	θ_max_ = 26.8°, θ_min_ = 2.0°
ω scans	*h* = −9→9
Absorption correction: integration (X-RED32; Stoe & Cie, 2002)	*k* = −10→10
*T*_min_ = 0.872, *T*_max_ = 0.892	*l* = −26→26
24485 measured reflections	

### Refinement

**Table:**

Refinement on *F*^2^	Primary atom site location: structure-invariant direct methods
Least-squares matrix: full	Secondary atom site location: difference Fourier map
*R*[*F*^2^ > 2σ(*F*^2^)] = 0.091	Hydrogen site location: inferred from neighbouring sites
*wR*(*F*^2^) = 0.307	H-atom parameters constrained
*S* = 1.36	*w* = 1/[σ^2^(*F*_o_^2^) + (0.2*P*)^2^] where *P* = (*F*_o_^2^ + 2*F*_c_^2^)/3
5417 reflections	(Δ/σ)_max_ < 0.001
328 parameters	Δρ_max_ = 2.04 e Å^−^^3^
0 restraints	Δρ_min_ = −1.13 e Å^−^^3^

### Special details

**Table:**

Geometry. Bond distances, angles *etc*. have been calculated using the rounded fractional coordinates. All su's are estimated from the variances of the (full) variance-covariance matrix. The cell e.s.d.'s are taken into account in the estimation of distances, angles and torsion angles
Refinement. Refinement on *F*^2^ for ALL reflections except those flagged by the user for potential systematic errors. Weighted *R*-factors *wR* and all goodnesses of fit *S* are based on *F*^2^, conventional *R*-factors *R* are based on *F*, with *F* set to zero for negative *F*^2^. The observed criterion of *F*^2^ > σ(*F*^2^) is used only for calculating -*R*-factor-obs *etc*. and is not relevant to the choice of reflections for refinement. *R*-factors based on *F*^2^ are statistically about twice as large as those based on *F*, and *R*-factors based on ALL data will be even larger.

### Fractional atomic coordinates and isotropic or equivalent isotropic displacement parameters (Å^2^)

**Table:**

	*x*	*y*	*z*	*U*_iso_*/*U*_eq_	
S1	0.03368 (13)	0.63784 (11)	0.12882 (4)	0.0578 (3)	
S2	−0.29088 (17)	0.44287 (14)	0.45285 (5)	0.0773 (4)	
O1	−0.0694 (5)	0.7915 (3)	0.11138 (12)	0.0735 (10)	
O2	0.2311 (4)	0.6020 (4)	0.11655 (14)	0.0790 (10)	
O3	0.0077 (4)	0.6048 (3)	0.20349 (10)	0.0617 (8)	
O4	−0.6871 (3)	0.6854 (3)	0.33989 (14)	0.0648 (9)	
N1	−0.3988 (3)	0.5354 (3)	0.31787 (12)	0.0412 (7)	
C1	−0.0691 (5)	0.4960 (4)	0.09658 (15)	0.0562 (10)	
C2	0.0407 (6)	0.3512 (5)	0.0825 (2)	0.0755 (16)	
C3	−0.0429 (8)	0.2421 (6)	0.0565 (3)	0.0875 (17)	
C4	−0.2337 (7)	0.2725 (5)	0.04587 (19)	0.0735 (13)	
C5	−0.3386 (6)	0.4142 (6)	0.0618 (2)	0.0726 (14)	
C6	−0.2604 (5)	0.5268 (5)	0.08621 (18)	0.0635 (11)	
C7	−0.3220 (11)	0.1501 (8)	0.0164 (3)	0.103 (2)	
C8	−0.1329 (4)	0.7120 (3)	0.23876 (13)	0.0446 (8)	
C9	−0.1186 (3)	0.6570 (3)	0.30901 (12)	0.0351 (7)	
C10	−0.2305 (3)	0.7881 (3)	0.34681 (12)	0.0359 (7)	
C11	−0.1458 (4)	0.9049 (3)	0.36804 (15)	0.0451 (8)	
C12	−0.2536 (6)	1.0252 (4)	0.40202 (17)	0.0569 (10)	
C13	−0.4427 (5)	1.0301 (4)	0.41386 (17)	0.0585 (10)	
C14	−0.5271 (4)	0.9165 (4)	0.39273 (17)	0.0532 (9)	
C15	−0.4213 (4)	0.7938 (3)	0.35887 (13)	0.0397 (7)	
C16	−0.5138 (4)	0.6687 (4)	0.33822 (14)	0.0430 (8)	
C17	−0.1943 (3)	0.5055 (3)	0.32432 (12)	0.0360 (7)	
C18	−0.1405 (3)	0.4301 (3)	0.38941 (13)	0.0376 (7)	
C19	0.0641 (3)	0.3302 (3)	0.40474 (9)	0.0283 (6)	
C20	0.0312 (6)	0.2882 (5)	0.47633 (19)	0.0633 (11)	
C21	−0.1398 (6)	0.3405 (4)	0.50120 (17)	0.0648 (13)	
C22	−0.4795 (4)	0.3999 (4)	0.30376 (15)	0.0495 (9)	
C23	−0.4857 (6)	0.3814 (5)	0.23322 (17)	0.0619 (11)	
C24	−0.5652 (5)	0.2386 (4)	0.21943 (15)	0.0513 (9)	
C25	−0.4571 (6)	0.1171 (5)	0.1868 (2)	0.0683 (12)	
C26	−0.5350 (8)	−0.0092 (5)	0.1723 (3)	0.0870 (18)	
C27	−0.7133 (8)	−0.0202 (5)	0.1926 (3)	0.090 (2)	
C28	−0.8215 (7)	0.1004 (7)	0.2275 (3)	0.092 (2)	
C29	−0.7476 (6)	0.2293 (5)	0.2385 (2)	0.0688 (14)	
H2	0.16880	0.32830	0.09050	0.0910*	
H3	0.03060	0.14590	0.04590	0.1050*	
H5	−0.46770	0.43490	0.05570	0.0870*	
H6	−0.33490	0.62340	0.09580	0.0760*	
H7A	−0.35930	0.18720	−0.02620	0.1540*	
H7B	−0.43050	0.13350	0.04180	0.1540*	
H7C	−0.23190	0.05260	0.01500	0.1540*	
H8A	−0.25770	0.71120	0.22420	0.0540*	
H8B	−0.11040	0.81860	0.23240	0.0540*	
H9	0.01370	0.63840	0.32080	0.0420*	
H11	−0.01820	0.90240	0.35960	0.0540*	
H12	−0.19770	1.10280	0.41680	0.0680*	
H13	−0.51390	1.11130	0.43640	0.0700*	
H14	−0.65510	0.92090	0.40090	0.0640*	
H17	−0.13800	0.43030	0.29270	0.0430*	
H19	0.17110	0.30490	0.37840	0.0340*	
H20	0.12680	0.22890	0.50100	0.0760*	
H21	−0.17070	0.31930	0.54400	0.0780*	
H22A	−0.60630	0.41290	0.32220	0.0590*	
H22B	−0.40510	0.30460	0.32370	0.0590*	
H23A	−0.35940	0.37080	0.21450	0.0740*	
H23B	−0.56290	0.47550	0.21340	0.0740*	
H25	−0.33280	0.11990	0.17470	0.0820*	
H26	−0.46430	−0.08790	0.14830	0.1040*	
H27	−0.76280	−0.10670	0.18340	0.1070*	
H28	−0.94180	0.09300	0.24300	0.1100*	
H29	−0.82200	0.31230	0.25920	0.0830*	

### Atomic displacement parameters (Å^2^)

**Table:**

	*U*^11^	*U*^22^	*U*^33^	*U*^12^	*U*^13^	*U*^23^
S1	0.0696 (6)	0.0664 (6)	0.0362 (4)	−0.0129 (4)	0.0092 (3)	−0.0030 (3)
S2	0.0886 (8)	0.0798 (7)	0.0602 (6)	−0.0132 (5)	0.0112 (5)	−0.0016 (5)
O1	0.109 (2)	0.0652 (16)	0.0445 (13)	−0.0169 (14)	0.0046 (13)	0.0039 (11)
O2	0.0700 (16)	0.110 (2)	0.0585 (16)	−0.0238 (15)	0.0145 (13)	−0.0087 (15)
O3	0.0799 (16)	0.0613 (13)	0.0357 (11)	0.0033 (11)	0.0104 (10)	−0.0042 (9)
O4	0.0301 (10)	0.0819 (17)	0.0867 (18)	−0.0192 (10)	0.0030 (10)	−0.0149 (14)
N1	0.0336 (10)	0.0471 (12)	0.0478 (13)	−0.0179 (9)	−0.0014 (9)	−0.0082 (10)
C1	0.0664 (19)	0.0632 (18)	0.0348 (13)	−0.0045 (15)	0.0080 (13)	−0.0060 (12)
C2	0.070 (2)	0.077 (3)	0.074 (3)	0.0044 (19)	−0.0009 (19)	−0.023 (2)
C3	0.094 (3)	0.076 (3)	0.086 (3)	0.007 (2)	−0.009 (2)	−0.027 (2)
C4	0.100 (3)	0.078 (2)	0.0468 (18)	−0.028 (2)	0.0008 (18)	−0.0057 (17)
C5	0.061 (2)	0.098 (3)	0.057 (2)	−0.0111 (19)	−0.0052 (16)	−0.0051 (19)
C6	0.065 (2)	0.067 (2)	0.0523 (18)	0.0027 (16)	−0.0040 (15)	−0.0067 (15)
C7	0.141 (5)	0.107 (4)	0.074 (3)	−0.051 (4)	−0.013 (3)	−0.017 (3)
C8	0.0441 (13)	0.0495 (14)	0.0388 (14)	−0.0091 (11)	0.0072 (11)	0.0015 (11)
C9	0.0308 (10)	0.0401 (12)	0.0361 (12)	−0.0118 (9)	0.0023 (9)	−0.0035 (9)
C10	0.0378 (12)	0.0361 (12)	0.0359 (12)	−0.0130 (9)	−0.0006 (9)	−0.0004 (9)
C11	0.0488 (14)	0.0408 (13)	0.0491 (15)	−0.0170 (11)	−0.0061 (12)	−0.0009 (11)
C12	0.082 (2)	0.0363 (13)	0.0566 (18)	−0.0194 (14)	−0.0076 (16)	−0.0060 (12)
C13	0.077 (2)	0.0389 (14)	0.0564 (18)	−0.0045 (14)	0.0073 (16)	−0.0088 (13)
C14	0.0483 (15)	0.0509 (16)	0.0569 (18)	−0.0030 (12)	0.0096 (13)	−0.0064 (13)
C15	0.0369 (12)	0.0394 (13)	0.0427 (13)	−0.0078 (10)	0.0009 (10)	−0.0020 (10)
C16	0.0323 (12)	0.0529 (15)	0.0464 (14)	−0.0140 (10)	−0.0004 (10)	−0.0055 (12)
C17	0.0341 (11)	0.0405 (12)	0.0360 (12)	−0.0132 (9)	0.0024 (9)	−0.0066 (9)
C18	0.0398 (12)	0.0363 (12)	0.0384 (13)	−0.0117 (9)	0.0022 (10)	−0.0041 (9)
C19	0.0316 (10)	0.0348 (11)	0.0186 (9)	−0.0109 (8)	−0.0072 (7)	0.0149 (8)
C20	0.073 (2)	0.0623 (19)	0.0561 (19)	−0.0200 (16)	−0.0158 (16)	0.0135 (15)
C21	0.102 (3)	0.0588 (19)	0.0415 (16)	−0.0379 (19)	0.0130 (17)	−0.0047 (14)
C22	0.0547 (16)	0.0554 (16)	0.0473 (15)	−0.0309 (13)	−0.0040 (12)	−0.0044 (12)
C23	0.085 (2)	0.065 (2)	0.0466 (17)	−0.0408 (18)	−0.0047 (16)	−0.0017 (15)
C24	0.0588 (17)	0.0537 (16)	0.0467 (16)	−0.0195 (13)	−0.0095 (13)	−0.0098 (13)
C25	0.066 (2)	0.074 (2)	0.065 (2)	−0.0102 (17)	−0.0058 (17)	−0.0131 (18)
C26	0.115 (4)	0.054 (2)	0.091 (3)	−0.002 (2)	−0.018 (3)	−0.032 (2)
C27	0.112 (4)	0.058 (2)	0.110 (4)	−0.036 (2)	−0.027 (3)	−0.014 (2)
C28	0.084 (3)	0.102 (4)	0.106 (4)	−0.057 (3)	−0.009 (3)	−0.012 (3)
C29	0.064 (2)	0.068 (2)	0.081 (3)	−0.0231 (17)	0.0036 (18)	−0.026 (2)

### Geometric parameters (Å, °)

**Table:**

S1—O1	1.422 (3)	C24—C25	1.383 (5)
S1—O2	1.418 (3)	C24—C29	1.381 (6)
S1—O3	1.583 (2)	C25—C26	1.387 (7)
S1—C1	1.749 (4)	C26—C27	1.363 (8)
S2—C18	1.678 (3)	C27—C28	1.403 (8)
S2—C21	1.613 (4)	C28—C29	1.371 (7)
O3—C8	1.448 (4)	C2—H2	0.9300
O4—C16	1.235 (4)	C3—H3	0.9300
N1—C16	1.364 (4)	C5—H5	0.9300
N1—C17	1.464 (3)	C6—H6	0.9300
N1—C22	1.468 (4)	C7—H7A	0.9600
C1—C2	1.387 (5)	C7—H7B	0.9600
C1—C6	1.383 (5)	C7—H7C	0.9600
C2—C3	1.376 (7)	C8—H8A	0.9700
C3—C4	1.381 (8)	C8—H8B	0.9700
C4—C5	1.363 (7)	C9—H9	0.9800
C4—C7	1.524 (8)	C11—H11	0.9300
C5—C6	1.363 (6)	C12—H12	0.9300
C8—C9	1.520 (4)	C13—H13	0.9300
C9—C10	1.508 (4)	C14—H14	0.9300
C9—C17	1.532 (3)	C17—H17	0.9800
C10—C11	1.393 (4)	C19—H19	0.9300
C10—C15	1.387 (4)	C20—H20	0.9300
C11—C12	1.390 (5)	C21—H21	0.9300
C12—C13	1.375 (6)	C22—H22A	0.9700
C13—C14	1.368 (5)	C22—H22B	0.9700
C14—C15	1.397 (4)	C23—H23A	0.9700
C15—C16	1.484 (4)	C23—H23B	0.9700
C17—C18	1.506 (4)	C25—H25	0.9300
C18—C19	1.594 (3)	C26—H26	0.9300
C19—C20	1.541 (4)	C27—H27	0.9300
C20—C21	1.328 (6)	C28—H28	0.9300
C22—C23	1.504 (5)	C29—H29	0.9300
C23—C24	1.518 (6)		
			
O1—S1—O2	119.4 (2)	C3—C2—H2	121.00
O1—S1—O3	108.65 (15)	C2—C3—H3	119.00
O1—S1—C1	109.86 (18)	C4—C3—H3	119.00
O2—S1—O3	104.97 (17)	C4—C5—H5	119.00
O2—S1—C1	109.28 (18)	C6—C5—H5	119.00
O3—S1—C1	103.47 (15)	C1—C6—H6	120.00
C18—S2—C21	94.67 (18)	C5—C6—H6	120.00
S1—O3—C8	119.2 (2)	C4—C7—H7A	109.00
C16—N1—C17	122.1 (2)	C4—C7—H7B	109.00
C16—N1—C22	119.8 (2)	C4—C7—H7C	109.00
C17—N1—C22	116.4 (2)	H7A—C7—H7B	109.00
S1—C1—C2	120.0 (3)	H7A—C7—H7C	110.00
S1—C1—C6	120.1 (3)	H7B—C7—H7C	109.00
C2—C1—C6	119.9 (4)	O3—C8—H8A	110.00
C1—C2—C3	118.9 (4)	O3—C8—H8B	110.00
C2—C3—C4	121.4 (5)	C9—C8—H8A	110.00
C3—C4—C5	118.3 (4)	C9—C8—H8B	110.00
C3—C4—C7	120.2 (5)	H8A—C8—H8B	109.00
C5—C4—C7	121.5 (5)	C8—C9—H9	109.00
C4—C5—C6	122.1 (4)	C10—C9—H9	109.00
C1—C6—C5	119.4 (4)	C17—C9—H9	109.00
O3—C8—C9	107.6 (2)	C10—C11—H11	120.00
C8—C9—C10	107.4 (2)	C12—C11—H11	120.00
C8—C9—C17	112.4 (2)	C11—C12—H12	120.00
C10—C9—C17	109.79 (19)	C13—C12—H12	120.00
C9—C10—C11	120.9 (2)	C12—C13—H13	120.00
C9—C10—C15	119.1 (2)	C14—C13—H13	120.00
C11—C10—C15	120.0 (2)	C13—C14—H14	120.00
C10—C11—C12	119.4 (3)	C15—C14—H14	120.00
C11—C12—C13	120.3 (3)	N1—C17—H17	108.00
C12—C13—C14	120.6 (3)	C9—C17—H17	108.00
C13—C14—C15	120.1 (3)	C18—C17—H17	108.00
C10—C15—C14	119.6 (3)	C18—C19—H19	131.00
C10—C15—C16	121.0 (2)	C20—C19—H19	131.00
C14—C15—C16	119.4 (3)	C19—C20—H20	122.00
O4—C16—N1	122.1 (3)	C21—C20—H20	122.00
O4—C16—C15	121.0 (3)	S2—C21—H21	122.00
N1—C16—C15	117.0 (3)	C20—C21—H21	122.00
N1—C17—C9	110.8 (2)	N1—C22—H22A	109.00
N1—C17—C18	110.7 (2)	N1—C22—H22B	109.00
C9—C17—C18	112.6 (2)	C23—C22—H22A	109.00
S2—C18—C17	123.12 (18)	C23—C22—H22B	109.00
S2—C18—C19	113.48 (18)	H22A—C22—H22B	108.00
C17—C18—C19	123.4 (2)	C22—C23—H23A	109.00
C18—C19—C20	98.8 (2)	C22—C23—H23B	109.00
C19—C20—C21	117.0 (3)	C24—C23—H23A	109.00
S2—C21—C20	116.0 (3)	C24—C23—H23B	109.00
N1—C22—C23	112.6 (3)	H23A—C23—H23B	108.00
C22—C23—C24	111.9 (3)	C24—C25—H25	120.00
C23—C24—C25	120.5 (3)	C26—C25—H25	120.00
C23—C24—C29	120.4 (3)	C25—C26—H26	119.00
C25—C24—C29	119.1 (4)	C27—C26—H26	119.00
C24—C25—C26	119.6 (4)	C26—C27—H27	120.00
C25—C26—C27	121.2 (5)	C28—C27—H27	120.00
C26—C27—C28	119.3 (5)	C27—C28—H28	120.00
C27—C28—C29	119.2 (5)	C29—C28—H28	120.00
C24—C29—C28	121.5 (4)	C24—C29—H29	119.00
C1—C2—H2	120.00	C28—C29—H29	119.00
			
O1—S1—O3—C8	11.0 (3)	C10—C9—C17—N1	50.8 (3)
O2—S1—O3—C8	139.7 (3)	C8—C9—C10—C11	−90.2 (3)
C1—S1—O3—C8	−105.8 (3)	C8—C9—C17—C18	166.7 (2)
O1—S1—C1—C2	152.1 (3)	C8—C9—C17—N1	−68.7 (3)
O2—S1—C1—C2	19.3 (4)	C9—C10—C11—C12	179.1 (3)
O3—S1—C1—C2	−92.1 (3)	C15—C10—C11—C12	0.9 (4)
O1—S1—C1—C6	−29.2 (3)	C11—C10—C15—C14	−0.5 (4)
O2—S1—C1—C6	−161.9 (3)	C9—C10—C15—C16	3.3 (4)
O3—S1—C1—C6	86.7 (3)	C11—C10—C15—C16	−178.4 (3)
C21—S2—C18—C19	0.4 (2)	C9—C10—C15—C14	−178.7 (3)
C18—S2—C21—C20	0.0 (3)	C10—C11—C12—C13	−0.9 (5)
C21—S2—C18—C17	−179.7 (2)	C11—C12—C13—C14	0.4 (5)
S1—O3—C8—C9	−176.8 (2)	C12—C13—C14—C15	0.1 (5)
C17—N1—C16—C15	8.5 (4)	C13—C14—C15—C10	−0.1 (5)
C17—N1—C16—O4	−171.1 (3)	C13—C14—C15—C16	178.0 (3)
C22—N1—C16—O4	−6.6 (4)	C10—C15—C16—O4	−168.7 (3)
C16—N1—C17—C18	85.2 (3)	C14—C15—C16—N1	−166.3 (3)
C17—N1—C22—C23	−89.1 (3)	C14—C15—C16—O4	13.4 (4)
C16—N1—C17—C9	−40.4 (3)	C10—C15—C16—N1	11.7 (4)
C22—N1—C16—C15	173.0 (3)	N1—C17—C18—C19	158.4 (2)
C16—N1—C22—C23	105.6 (3)	N1—C17—C18—S2	−21.5 (3)
C22—N1—C17—C18	−79.8 (3)	C9—C17—C18—C19	−77.0 (3)
C22—N1—C17—C9	154.6 (2)	C9—C17—C18—S2	103.1 (2)
C6—C1—C2—C3	1.9 (6)	S2—C18—C19—C20	−0.6 (3)
C2—C1—C6—C5	−0.4 (6)	C17—C18—C19—C20	179.5 (3)
S1—C1—C2—C3	−179.3 (4)	C18—C19—C20—C21	0.6 (4)
S1—C1—C6—C5	−179.2 (3)	C19—C20—C21—S2	−0.4 (5)
C1—C2—C3—C4	−1.8 (8)	N1—C22—C23—C24	178.7 (3)
C2—C3—C4—C5	0.0 (8)	C22—C23—C24—C25	−118.9 (4)
C2—C3—C4—C7	179.4 (5)	C22—C23—C24—C29	62.1 (5)
C7—C4—C5—C6	−177.8 (4)	C23—C24—C25—C26	−177.3 (4)
C3—C4—C5—C6	1.6 (7)	C29—C24—C25—C26	1.8 (6)
C4—C5—C6—C1	−1.4 (6)	C23—C24—C29—C28	−178.9 (4)
O3—C8—C9—C17	−72.4 (3)	C25—C24—C29—C28	2.0 (6)
O3—C8—C9—C10	166.8 (2)	C24—C25—C26—C27	−3.6 (8)
C8—C9—C10—C15	88.0 (3)	C25—C26—C27—C28	1.5 (9)
C10—C9—C17—C18	−73.8 (2)	C26—C27—C28—C29	2.2 (9)
C17—C9—C10—C11	147.2 (2)	C27—C28—C29—C24	−4.0 (8)
C17—C9—C10—C15	−34.5 (3)		

### Hydrogen-bond geometry (Å, °)

**Table:**

*D*—H···*A*	*D*—H	H···*A*	*D*···*A*	*D*—H···*A*
C9—H9···O4^i^	0.98	2.34	3.285 (3)	161
C12—H12···Cg1^ii^	0.93	2.65	3.567 (4)	168

Symmetry codes: (i) *x*+1, *y*, *z*; (ii) *x*, *y*+1, *z*.
